# Nasal Catarrh and Headache

**Published:** 1905-11

**Authors:** 


### Nasal Catarrh and Headache.

NASAL catarrh, both acute and chronic, frequently serves as the cause of headache. The pain is generally one of persistent type classed as congestive. Examination in these cases may show suppuration of accessory sinuses with marked nasal obstruction due to small spurs, deviated septum and general hypertrophy. These obstructions, however, may prove of little import if the engorged membrane can be readily depleted and the local circulatory system restored. Frequently this can be accomplished by instructing the patient in the use of glyco-thymoline in a 25-per cent. warm solution by means of the K. & O. nasal douche. Glyco-thymoline by its hygroscopic property stimulates capillary circulation and restores normal glandular action, promptly allaying those symptoms dependent on congestion. The solution should be applied at least twice daily until the nasal membrane is found to be perfectly normal.

A CHICAGO physician says that automobiling is a cure for indigestion. Sure. Anyone who is fairly hit by an automobile is never again troubled by indigestion or, for that matter, any of the other ills to which man is heir.—*"John Smith" in Buffalo Express.*

